# PAIN MANAGEMENT IN DEMENTIA: A NEED FOR SPECIFICITY AND SUSTAINABILITY IN EXERCISE-BASED PROGRAMS

**DOI:** 10.1093/geroni/igae098.4345

**Published:** 2024-12-31

**Authors:** Annalisa Na, Joke Bradt, Julie Fritz, Gediminas Gliebus, Laura Gitlin

**Affiliations:** Drexel University, Drexel University, Pennsylvania, United States; Drexel University, Philadelphia, Pennsylvania, United States; University of Utah, Salt Lake City, Utah, United States; Baptist Health South Florida, Boca Raton, Florida, United States; Drexel University, Philadelphia, Pennsylvania, United States

## Abstract

Over 50% of people living with dementia (PLWD) experience pain associated with functional decline. Pain and dementia are heterogeneous and complex, making the development of effective nonpharmacological interventions challenging. Hence, we used Intervention Mapping (IM), an evidence-based process for designing complex behavioral interventions. This study focuses on completing Step 1 of IM (needs assessment) to define population and intervention priorities. We conducted and synthesized the findings of three studies: (1) a scoping review of 81 studies summarizing current literature, (2) an analysis of the National Health and Aging Trends Study (NHATS) database (n=9,974) to characterize pain among community-dwelling PLWD, (3) qualitative interviews with clinicians (n=19) and PLWD/caregivers (n=17) to assess perspectives on current care. The scoping review revealed that most research focuses on passive interventions in long-term care facilities and lacks specificity in reporting pain diagnosis and dementia severity. From the NHATS database, low back and knee osteoarthritis were the most prevalent pain conditions among community-dwelling PLWD. Qualitative analysis of interviews confirmed scoping review findings of an overall lack of exercise-based interventions for pain despite being standard care among cognitively intact individuals. While there was a shared interest in exercise-based interventions, clinicians expressed concerns about adapting and sustaining these programs. Triangulating data (epidemiological and qualitative sources) and following IM provides foundational support to design clinical interventions for pain in PLWD. Specifically, intervention development should prioritize community-dwelling PLWD with knee and/or low back pain using therapeutic exercises tailored to the individual’s interests and abilities; however, research is needed to address sustainability.

